# Association between alcohol consumption and symptom severity and quality of life in patients with fibromyalgia

**DOI:** 10.1186/ar4200

**Published:** 2013-03-15

**Authors:** Chul H Kim, Ann Vincent, Daniel J Clauw, Connie A Luedtke, Jeffrey M Thompson, Terry D Schneekloth, Terry H Oh

**Affiliations:** 1 Department of Physical Medicine and Rehabilitation, Mayo Clinic, Rochester, MN 55905, USA; 2 Department of Rehabilitation Medicine, Kyungpook National University Hospital, Daegu, 700721 Korea; 3 Division of General Internal Medicine Mayo Clinic, Rochester, MN 55905, USA; 4 Department of Anesthesiology, Medicine, and Psychiatry, University of Michigan, Ann Arbor, MI 48109, USA; 5 Department of Nursing Mayo Clinic, Rochester, MN 55905, USA; 6 Department of Psychiatry and Psychology, Mayo Clinic, Rochester, MN 55905, USA

## Abstract

**Introduction:**

Although alcohol consumption is a common lifestyle behavior with previous studies reporting positive effects of alcohol on chronic pain and rheumatoid arthritis, no studies to this date have examined alcohol consumption in patients with fibromyalgia. We examined the association between alcohol consumption and symptom severity and quality of life (QOL) in patients with fibromyalgia.

**Methods:**

Data on self-reported alcohol consumption from 946 patients were analyzed. Subjects were grouped by level of alcohol consumption (number of drinks/week): none, low (≤3), moderate (>3 to 7), and heavy (>7).

Univariate analyses were used to find potential confounders, and analysis of covariance was used to adjust for these confounders. Tukey HSD pairwise comparisons were used to determine differences between alcohol groups.

**Results:**

Five hundred and forty-six subjects (58%) did not consume alcohol. Low, moderate, and heavy levels of alcohol consumption were reported for 338 (36%), 31 (3%), and 31 patients (3%), respectively. Employment status (*P *<0.001), education level (*P *= 0.009), body mass index (*P *= 0.002) and opioid use (*P *= 0.002) differed significantly among groups with drinkers having higher education, a lower BMI, and a lower frequency of unemployment and opioid use than nondrinkers. After adjusting for these differences, the measures including the number of tender points (*P *= 0.01), FIQ total score (*P *= 0.01), physical function (*P *<0.001), work missed (*P *= 0.005), job ability (*P *= 0.03), and pain (*P *= 0.001) differed across groups, as did the SF-36 subscales of physical functioning (*P *<0.001), pain index (*P = *0.002), general health perception (*P *= 0.02), social functioning (*P *= 0.02), and the physical component summary (*P *<0.001). Pairwise comparison among the 4 groups showed that the moderate and low alcohol drinkers had lower severity of fibromyalgia symptoms and better physical QOL than nondrinkers.

**Conclusions:**

Our study demonstrates that low and moderate alcohol consumption was associated with lower fibromyalgia symptoms and better QOL compared to no alcohol consumption. The reasons for these results are unclear. Since recent studies have demonstrated that γ-Aminobutyric Acid (GABA) levels are low in fibromyalgia, and alcohol is known to be a GABA-agonist, future studies should examine whether alcohol could have a salutary effect on pain and other symptoms in fibromyalgia.

## Introduction

Fibromyalgia is a chronic condition characterized by widespread pain, tenderness, and decreased pain threshold to pressure and other stimuli [1,2]. It is often associated with fatigue, unrefreshing sleep, and cognitive symptoms as well as multiple other somatic symptoms including headache, irritable bowel and bladder symptoms, and depression [3]. Although the cause of fibromyalgia is unclear, extensive research has helped understanding of fibromyalgia and indicates that fibromyalgia is a central amplification disorder of pain perception resulting from neurochemical imbalances in the central nervous system [4-6].

Alcohol consumption has both harmful and beneficial effects on health. On the one hand, drinking large amounts of alcohol for years is hazardous [7]. On the other hand, epidemiologic and clinical evidence suggests that moderate drinking is associated with a reduced risk of cardiovascular disease [8] and ischemic stroke [9]. In US adults, moderate alcohol consumption is considered a lifestyle behavior that supports cardiovascular health, in conjunction with a prudent diet, regular physical activity, a healthy weight, and not smoking [10]. Furthermore, regular low-to-moderate alcohol consumption has been associated with better quality of life (QOL), mood, and subjective health in studies of young and older adults [11-16] and a protective factor for better health in a general population with and without chronic musculoskeletal pain [17,18].

Studies on alcohol consumption in chronic pain have shown conflicting results. Whereas a systematic review showed no positive association or dose response between alcohol consumption and low back pain [19], individual studies of men with back pain disability showed that those who consumed large amounts of alcohol had less physical disability, despite similar pain [20], and those who consumed alcohol regularly were less likely to have associated chronic pain [21]. Epidemiologic studies on alcohol and rheumatoid arthritis have reported interesting results, namely that moderate alcohol consumption may decrease the risk of rheumatoid arthritis development, severity and progression, possibly by reducing levels of some inflammatory markers [22-24].

Although alcohol consumption is a common lifestyle behavior, no studies to date have examined alcohol consumption in the setting of fibromyalgia. The objective of this study was to evaluate the association between alcohol consumption and symptom severity and QOL in patients with fibromyalgia.

## Materials and methods

### Subjects

This study was approved by the Mayo Clinic Institutional Review Board, and all patients provided written consent to participate in the study. This is a cross-sectional study using data of patients previously described [25]. Participants were seen in the Fibromyalgia Treatment Program (FTP) during the study period from May 1, 2001, through April 30, 2004, and had the confirmed diagnosis of fibromyalgia according to the 1990 American College of Rheumatology criteria [1]. All patients were older than 18 years, had history of alcohol consumption in the electronic medical record, and had completed the Fibromyalgia Impact Questionnaire (FIQ) [26] and the Short Form-36 Health Survey (SF-36) [27,28] at the time of evaluation in the FTP. All participants underwent a standardized evaluation, including a tender-point assessment by a registered nurse. Demographic and social variables and the number of tender points were abstracted from the medical record.

### Alcohol consumption determination and grouping

Alcohol consumption data (self-reported alcohol history, taken as a part of the social history) were collected from the electronic medical record. If patients were current alcohol users, they were asked specifically about the frequency of use per week or month, the number of drinks consumed per each time, and the type of beverage consumed. One drink was defined as 12 fluid ounces of regular beer (5% alcohol), 5 fluid ounces of wine (12% alcohol), or 1.5 fluid ounces of distilled spirits (40% alcohol) [29]. We calculated drinks per week by multiplying the frequency of use per week with the number of drinks per each time. Subjects were grouped by the level of alcohol consumption (number of drinks per week): none, low (≤3), moderate (>3 to 7), and heavy (>7), consistent with guidelines from the National Institute on Alcohol Abuse and Alcoholism, which distinguish between low-risk and at-risk consumption patterns [29]. We did not differentiate on the basis of beverage type for this study.

### Fibromyalgia-related symptoms and QOL assessment

Participants completed the FIQ and the SF-36 at the time of their evaluation in the FTP. The FIQ was first published in 1991 and was designed to assess health status specifically in patients with fibromyalgia [26]. It is a self-administered questionnaire that measures multiple domains of fibromyalgia symptoms and functional impairment, and it contains 20 questions that assess the following areas: physical functioning, overall well-being in the previous week, days of work missed, and symptoms of pain, fatigue, morning tiredness, stiffness, job difficulty, anxiety, and depression. Possible scores range from 0 to 100, with a high score indicating a greater impact of fibromyalgia-related symptomatology [26]. The FIQ was scored according to the directions outlined by Bennett [30].

The SF-36 consists of 36 questions to assess health-related QOL. It was developed as a potential tool for monitoring patient outcomes in a busy clinical setting [27,28]. It is a self-administered questionnaire that measures eight health concepts: physical functioning, role limitation due to physical health, pain index, general health perception, vitality score, social functioning, role limitations due to emotional health, and mental health. It also provides physical composite and mental composite scores. The SF-36 score can range from 0 to 100, with higher scores indicating better health status [28].

### Statistical analysis

Demographic and social characteristics were compared across alcohol consumption groups using one-way analysis of variance (ANOVA) for continuous variables and chi square (χ^2^) tests for categorical variables. Unadjusted comparisons of the numeric rating scale for pain, number of tender points, and FIQ and SF-36 scores across groups were performed using one-way ANOVA. Analysis of covariance (ANCOVA) was used to adjust comparisons for potential confounders across the four alcohol groups. For the multivariate ANCOVA models, potential confounders were selected based on either being clinically important (age) or showing significance from univariate analyses (employment status, education level, body mass index (BMI), and opioid use). Univariate collinearity evaluations were done to rule out correlated covariates. None of the covariates were strongly correlated. The Tukey honestly significant difference (HSD) method was used to perform pairwise comparisons of least-squares means between alcohol consumption groups, adjusted for baseline differences, when the overall effect in the multivariable model was found to be statistically significant. *P *values <0.05 were considered statistically significant. Analysis was performed using JMP (version 8, SAS Institute Inc, Cary, NC, USA).

## Results

### Patients

Among the 988 patients seen during the study period, 42 patients had incomplete data on alcohol consumption and were excluded from further analysis. The final study subjects consisted of 946 fibromyalgia patients (893 women) with a mean age of 49 years (SD, 12.7). Patient characteristics, stratified by level of alcohol consumption, are shown in Table 1. Five hundred and forty-six subjects (58%) did not consume alcohol. Low, moderate, and heavy levels of alcohol consumption were reported for 338 (36%), 31 (3%), and 31 patients (3%), respectively. Employment status (*P *<0.001), education level (*P *= 0.009), (BMI) (*P *= 0.002), and opioid use (*P *= 0.002) differed significantly across the four groups, with drinkers having higher education, a lower BMI, and a lower frequency of unemployment and opioid use than nondrinkers (Table 1).

**Table 1:** Patient characteristics (number of patients = 946)

Characteristic	Alcohol consumption level^a^				*P*-value
		
	None (*n *= 546)	Low (*n *= 338)	Moderate (*n *= 31)	Heavy (*n *= 31)	
Female, n (%)	517 (94.7)	318 (94.1)	28 (90.3)	30 (96.8)	0.69
Age, y, mean (SD)	49 (13.1)	48 (12.0)	52 (12.3)	52 (14.3)	0.08
Race/ethnicity, n (%)					0.50
White	536 (98.2)	335 (99.1)	31 (100)	30 (96.8)	
Nonwhite	10 (1.8)	3 (0.9)	0 (0)	1 (3.2)	
Abuse history, n/total (%)	155/537 (28.9)	108/334 (32.3)	9 (29.0)	12 (38.7)	0.53
Current tobacco user, n/total (%)	80/543 (14.7)	45 (13.3)	7 (22.6)	5 (16.1)	0.55
Marital status, n (%)					0.90
Married	421 (77.1)	252 (74.6)	22 (71.0)	23 (74.2)	
Unmarried	114 (20.9)	78 (23.1)	8 (25.8)	8 (25.8)	
Widowed	11 (2.0)	8 (2.4)	1 (3.2)	0 (0)	
Employment status, n (%)					<0.001
Employed	255 (46.7)	213 (63.0)	20 (64.5)	14 (45.2)	
Homemaker	56 (10.3)	19 (5.6)	2 (6.5)	5 (16.1)	
Retired	70 (12.8)	33 (9.8)	5 (16.1)	6 (19.4)	
Unemployed	165 (30.2)	73 (21.6)	4 (12.9)	6 (19.4)	
Education level, n/total (%)					0.009
<12^th ^grade	24/538 (4.5)	6/334 (1.8)	1 (3.2)	2/30 (6.7)	
High school	188/538 (34.9)	89/334 (26.7)	9 (29.0)	7/30 (23.3)	
Some college or technical college	171/538 (31.8)	110/334 (32.9)	5 (16.1)	10/30 (33.3)	
College, graduate school	155/538 (28.8)	129/334 (38.6)	16 (51.6)	11/30 (36.7)	
Body mass index, kg/m^2^, mean (SD)	30.5 (7.4)	29.2 (7.2)	27.4 (6.0)	27.0 (4.3)	0.002
Opioid use, n/total (%)	155/545 (28.4)	67 (19.8)	2 (6.45)	6 (19.35)	0.002
Benzodiazepine use, n/total (%)	120/545 (22.0)	61 (18.1)	4 (12.9)	9 (29.0)	0.21
Alcoholic drinks consumed per week, mean number (SD)	...	0.5 (0.7)	5.8 (1.2)	14.8 (8.5)	...

**^a^**Alcohol consumption levels were defined as follows: none, 0 drinks/wk; low, ≤3 drinks/wk; moderate, >3 to 7 drinks/wk; heavy, >7 drinks/wk. n, number of patients.

### Comparison of symptoms and QOL among patient groups

After adjusting for age, employment status, education level, BMI and opioid use, we found significant group differences in the number of tender points (*P *= 0.01) (Table 2), FIQ total score (*P *= 0.01), FIQ subscales of physical function (*P *<0.001), work missed (*P *= 0.005), job ability (*P *= 0.03), and pain (*P *= 0.001) (Table 3), and SF-36 subscales of physical functioning (*P *<0.001), pain index (*P *= 0.002), general health perception (*P *= 0.02), social functioning (*P *= 0.02), and physical component summary (*P *<0.001) (Table 4). Pain scores were significant on unadjusted analysis and became not significant on adjusted analysis (Table 2). Moderate drinkers had overall lower fibromyalgia-related symptoms, and higher QOL scores than the other groups.

**Table 2:** Comparison of pain and tender points with alcohol use (number of patients = 946)

Characteristic	Alcohol consumption level^a^				*P*-value	Adjusted *P*-value^b^
			
	None (*n *= 546)	Low (*n *= 338)	Moderate (*n *= 31)	Heavy (*n *= 31)		
Pain score, mean (SD)^c^						
Now	6.2 (1.7)	5.8 (1.9)	5.4 (1.9)	5.5 (1.4)	0.003	0.11
Best	3.9 (1.9)	3.6 (1.8)	2.9 (1.6)	3.2 (1.7)	0.002	0.13
Worst	9.3 (1.6)	9.1 (1.6)	8.5 (1.7)	8.8 (1.8)	0.01	0.21
Tender points, mean number (SD)	16.2 (2.1)	16.3 (2.1)	14.9 (2.7)	15.3 (2.6)	0.001	0.01

^a^Alcohol consumption levels were defined as follows: none, 0 drinks/wk; low, ≤3 drinks/wk; moderate, >3 to 7 drinks/wk; heavy, >7 drinks/wk. ^b^After adjusting for age, employment status, education level, body mass index and opioid use. ^c^Pain score was evaluated using a numeric rating scale (range of possible scores, 1 to 10).

**Table 3:** Comparison of FIQ scores with alcohol use (number of patients = 946)

FIQ^a^	Alcohol consumption level^b^				*P*-value	Adjusted *P*-value^c^
			
	None (*n *= 546)	Low (*n *= 338)	Moderate (*n *= 31)	Heavy (*n *= 31)		
Total score	65.1 (17.0)	61.0 (15.9)	54.7 (17.1)	62.2 (15.8)	<0.001	0.01
Physical function	5.0 (2.2)	4.3 (2.3)	3.6 (2.3)	4.1 (2.4)	<0.001	<0.001
Feel good	7.9 (2.2)	7.8 (2.3)	6.7 (2.4)	7.5 (2.5)	0.03	0.10
Work missed	4.7 (3.6)	3.5 (3.5)	2.5 (3.2)	3.7 (3.6)	<0.001	0.005
Job ability	7.1 (2.4)	6.5 (2.4)	5.8 (2.1)	6.7 (2.3)	<0.001	0.03
Pain	7.3 (2.0)	7.0 (2.1)	5.6 (2.1)	7.1 (1.7)	<0.001	0.001
Fatigue	8.3 (2.0)	8.1 (2.0)	7.7 (2.0)	7.9 (2.3)	0.19	0.41
Morning tiredness	7.9 (2.3)	7.8 (2.3)	7.7 (2.1)	8.1 (1.9)	0.71	0.56
Stiffness	7.4 (2.3)	7.3 (2.3)	6.7 (2.4)	7.6 (1.9)	0.39	0.23
Depression	4.3 (3.3)	3.9 (3.1)	3.7 (2.9)	4.4 (3.2)	0.38	0.37
Anxiety	5.1 (3.1)	4.9 (3.0)	4.6 (3.2)	5.2 (2.7)	0.68	0.81

FIQ, Fibromyalgia Impact Questionnaire. ^a^All values are reported as mean (SD). ^b^Alcohol consumption levels were defined as follows: none, 0 drinks/wk; low, ≤3 drinks/wk; moderate, >3 to 7 drinks/wk; heavy, >7 drinks/wk. ^c^After adjusting for age, employment status, education level, body mass index and opioid use.

**Table 4:** Comparison of SF-36 Scores with alcohol use (number of patients = 946).

SF-36^a^	Alcohol consumption level^b^				*P*-value	Adjusted *P*-value^c^
			
	None (*n *= 546)	Low (*n *= 338)	Moderate (*n *= 31)	Heavy (*n *= 31)		
Physical functioning	35.5 (21.5)	42.4 (22.4)	51.1 (23.2)	51.5 (22.9)	<0.001	<0.001
Role-physical	7.0 (17.5)	9.5 (21.1)	11.3 (24.9)	8.1 (20.8)	0.18	0.32
Pain index	23.0 (14.5)	27.2 (14.9)	32.5 (12.9)	30.4 (15.1)	<0.001	0.002
General health perceptions	35.7 (20.0)	41.1 (21.0)	43.5 (18.2)	43.0 (23.6)	<0.001	0.02
Vitality	16.3 (15.6)	18.9 (17.0)	19.7 (17.0)	23.7 (15.8)	0.01	0.33
Social functioning	36.4 (25.0)	42.1 (24.3)	47.6 (24.5)	46.0 (25.9)	<0.001	0.02
Role-emotional	45.0 (42.9)	48.9 (42.7)	45.2 (41.8)	46.2 (41.9)	0.62	0.91
Mental health index	55.5 (22.1)	58.4 (20.0)	60.8 (20.0)	59.9 (18.9)	0.13	0.51
Physical component summary	25.6 (7.1)	27.9 (7.8)	30.6 (8.1)	30.1 (9.0)	<0.001	<0.001
Mental component summary	39.4 (12.0)	40.6 (11.4)	40.4 (11.0)	40.7 (12.4)	0.49	0.76

SF-36, Short Form-36 Health Survey. ^a^All values are reported as mean (SD). ^b^Alcohol consumption levels were defined as follows: none, 0 drinks/wk; low, ≤3 drinks/wk; moderate, >3 to 7 drinks/wk; heavy, >7 drinks/wk. ^c^After adjusting for age, employment status, education level, body mass index and opioid use.

Pairwise comparison among the four groups showed that the moderate and low alcohol drinkers had better physical QOL than nondrinkers, with significant differences for SF-36 physical functioning, pain index, and physical component summary scores (Table 5). Additionally, low drinkers had better scores of SF-36 general health perceptions and social functioning scales than nondrinkers. For heavy drinkers, the only significant finding was better scores on the SF-36 physical functioning scale than nondrinkers. Regarding the severity of fibromyalgia-related symptoms, the moderate drinkers had significantly lower FIQ pain scores than all other groups, better FIQ total scores than nondrinkers, better physical function than nondrinkers, and fewer tender points than low drinkers. Also, low drinkers had better scores on FIQ work missed, and on physical function than nondrinkers.

**Table 5:** Pairwise comparison among alcohol consumption groups

Characteristic	Pairwise comparison of outcomes among alcohol consumption groups^a^, *P*-value^b^					
	
	None vs low	None vs moderate	None vs heavy	Low vs moderate	Low vs heavy	Moderate vs heavy
Tender points	0.76	0.10	0.27	0.04	0.14	0.98
FIQ						
Total	0.11	0.049	0.98	0.32	0.61	0.13
Physical function	<0.001	0.04	0.76	0.65	0.96	0.60
Work missed	0.004	0.29	0.96	0.97	0.85	0.79
Job ability	0.10	0.11	0.98	0.51	0.97	0.53
Pain	0.61	<0.001	0.98	0.005	0.80	0.009
SF-36						
Physical functioning	0.047	0.006	0.01	0.11	0.19	1.00
Pain index	0.02	0.03	0.43	0.39	0.98	0.81
General health perceptions	0.01	0.58	0.84	1.00	0.99	0.99
Social functioning	0.03	0.28	0.86	0.88	0.99	0.88
Physical component summary	0.009	0.01	0.09	0.26	0.69	0.95

FIQ, Fibromyalgia Impact Questionnaire; SF-36, Short Form-36 Health Survey. ^a^Alcohol consumption levels were defined as follows: none, 0 drinks/wk; low, ≤3 drinks/wk; moderate, >3 to 7 drinks/wk; heavy, >7 drinks/wk. ^b^Tukey HSD method was used to perform pairwise comparisons of least-squares means between groups from a multivariate model that adjusted for age, employment status, education level, body mass index and opioid use. *P*-values <0.05 were considered statistically significant.

## Discussion

Our cross-sectional study demonstrates that for patients with fibromyalgia, low and moderate alcohol consumption was associated with lower fibromyalgia symptoms and better QOL compared to no alcohol consumption. Drinkers had higher education, a lower BMI and a lower frequency of unemployment and opioid use than nondrinkers. Among drinkers, moderate alcohol consumption was associated with lower FIQ pain scores than low and heavy drinkers, and a lower number of tender points than low drinkers even after adjusting for confounding covariates. The reasons for these results are unclear.

To our knowledge, no other reports on alcohol consumption, symptom severity, and QOL in patients with fibromyalgia have been published to date, which limits our ability to further validate our findings. However, in the general population, those who had low to moderate alcohol consumption had better QOL scores in physical and mental domains of SF-36 [12,14], especially in the physical domains [11,16]. Also, in a cross-sectional study [21] those who consumed alcohol regularly were less likely to have chronic pain, and regular alcohol consumption has been shown to be protective of better health in a general population with and without chronic musculoskeletal pain [17,18], as well as being associated with lower risk and severity of rheumatoid arthritis [23]. Our results were generally similar to these studies in terms of better QOL scores, especially for the physical domains, for the low and moderate drinkers.

It is notable that we did not observe the same association in heavy drinkers. The only significant finding in heavy drinkers as compared to nondrinkers was better scores on the SF-36 physical functioning scale. This finding suggests that there are other unknown factors or comorbid conditions affecting fibromyalgia symptoms and QOL. Clinical and epidemiological studies have suggested an association between excessive alcohol consumption and major depressive episodes [31]. Although our study was not meant to adequately assess depression, we did not observe significant differences in the FIQ depression nor SF-36 mental component summary scores between the groups. Binge drinking (five or more drinks on one occasion) is reported to be associated with many health problems, including worse QOL and mental distress [32,33]. There were five binge drinkers (16%) in our heavy drinker group, who had overall worse scores in the FIQ and SF-36 than non-bingeing heavy drinkers: mean FIQ total 74.5 (SD 17.1) vs 59.8 (14.7), SF-36 physical component summary 29.8 (7.9) vs 30.1(9.3) and SF-36 mental component summary 26.9 (4.5) vs 43.4 (11.7) in the bingeing vs non-bingeing heavy drinkers, respectively. However, the small number of patients limited further statistical analysis. It is possible that the worse scores in the binge-drinkers affected the overall outcome of heavy drinkers.

We speculated about possible mechanisms by which alcohol consumption attenuates fibromyalgia symptoms, thus resulting in improved QOL. We showed that low and moderate alcohol consumption in patients with fibromyalgia was associated with higher social status, as noted in other studies in terms of employment [34] and education [34-36], and favorable BMI in drinkers, as also noted elsewhere [35,37]. The 1999 to 2006 National Health and Nutrition Examination Survey in the United States [36] determined that the percentage of drinkers increased with higher education level, and current drinkers had a lower BMI than never drinkers or former drinkers. In our study, even after adjusting for social variables and BMI, we still found the same association that those with low or moderate alcohol consumption had fewer fibromyalgia symptoms and higher QOL than nondrinkers. Thus, socioeconomic status alone apparently does not explain the findings.

Studies suggest that fibromyalgia is a central amplification disorder of pain perception due to neurochemical imbalances in the central nervous system [4,6]. We believe that a more likely mechanism, in view of our finding that alcohol consumption attenuates fibromyalgia symptoms, might be centrally mediated via γ-Aminobutyric Acid (GABA) system. Previous studies have demonstrated that ethanol enhances GABA release in the central nervous system [38,39], and it is interesting that a recent study showed that GABA levels in the brain are decreased in patients with fibromyalgia [5]. Gamma-hydroxybutyrate is a metabolite of GABA and was found to be effective at reducing fibromyalgia symptoms but did not receive Food and Drug Administration approval for the management of fibromyalgia because of concerns about abuse [40,41]. Thus, increase in GABA production may represent a mechanism by which alcohol consumption decreases fibromyalgia symptoms.

It is uncertain whether the effect of alcohol on fibromyalgia symptoms and QOL might be mediated by its psychological benefits as a stress reliever or a factor associated with social integration [12,42]. Several studies showed that small doses of alcohol were associated with improved mood and decreased depression and tension [42,43]. Also, its favorable effects on cardiovascular health might influence QOL, particularly for the elderly [12]. Alcohol can suppress the synthesis of proinflammatory cytokines and chemokines such as tumor necrosis factor and interleukin-6, *in vivo *or *in vitro *(or both) [44,45], and it may also have a role as an anti-inflammatory factor. However, the association of cytokines with fibromyalgia is controversial [46], with several studies supporting a positive association [47,48], and another study showing no association [49]. Alcohol may provide short-term analgesic effect as demonstrated in a study by James *et al*. [50], in which alcohol significantly increased the pain threshold and decreased the perception of pain after an infusion of alcohol for 1 hour. In our study, moderate but not heavy drinkers, had significantly lower FIQ pain than all other groups, and it is unlikely that the short-term analgesic effect of alcohol explains the finding.

Alcohol use in our patients was 42%, with most of them being low-drinkers. The rate of alcohol use among our patients with fibromyalgia was lower than the 53% rate for women in the general population [10]. Our study was based on data from 2001 to 2004. A recent Gallup report showed that rates of alcohol consumption overall have stayed stable in spite of some yearly fluctuations (Gallup report 2012) [51]. Therefore, we believe our findings are still relevant. We observed significant group differences in opioid use with the lowest use in the moderate drinkers and the highest use in the nondrinkers, suggesting that the nondrinkers may have more severe symptoms than the other groups. We cannot rule out the possibility that the findings were biased by individuals who did not drink alcohol because of more severe symptoms of fibromyalgia. The 3% rate of heavy-drinkers (equivalent to more than one drink per day) in our study was also lower than the 7% rate for US women [10]. We do not know why the drinking rate was lower in our patient group but speculate that it might be related to 1) decreased social functioning due to chronic pain and therefore, fewer occasions to drink alcohol socially; 2) self-perceived chronic health concerns and lower QOL, leading to different drinking habits; 3) concerns of alcohol interacting with medications such as sedatives or narcotics, and 4) possible under-reporting of drinking.

We urge caution when generalizing the findings of this study because of the relatively small number of moderate and heavy drinkers in the study. Furthermore, we do not recommend that patients with fibromyalgia start or increase drinking for their symptoms. One study showed that alcohol abuse was one of the most frequent psychiatric problems in patients with chronic pain, and a significant portion of chronic pain patients had a history of alcohol abuse before the onset of their pain [52]. Therefore, our findings should be interpreted carefully.

This study has several limitations. First, the amount of alcohol consumption in this study was self-reported and thus may be biased because of possible under-reporting. However, simple self-administered questionnaires previously have provided useful estimates of alcohol intake [53,54]. Our patient population included four patients who were younger than 21 years (drinking reports: none (three patients), low (one patient)), which is the legal drinking age in the US. Our results were the same when we excluded them. Therefore, we kept them in the study. Second, we had small sample sizes for those with moderate and heavy alcohol consumption, and this limited the power to detect associations in the groups. Third, the cross-sectional design of the current study allowed us to say only that low-to-moderate alcohol consumption appeared to be associated with lower fibromyalgia symptoms and improved QOL. However, the reasons for, and the clinical importance of this association cannot be determined in this study, and the associations may be due to unmeasured confounding variables. Fourth, we did not use a validated specific questionnaire for depression, and our study was not meant to adequately assess depression. Therefore, we were not able to do further analysis on comorbid psychiatric diagnoses. Fifth, we did not differentiate former drinkers from abstainers. Previous studies have shown conflicting findings, ranging from worse QOL in former drinkers than abstainers [13,14], no difference in subjective health [55,56], and better QOL in former drinkers than abstainers [57]. However, this diversity in results might be due to variations in study design [8]. We also did not consider the beverage type consumed (for example, beer versus wine versus spirits) because the health effects of alcohol primarily have been attributed to ethanol content rather than to other components of each beverage type [58-60].

## Conclusions

Our findings suggest that low-to-moderate alcohol consumption was associated with lower fibromyalgia symptoms and better QOL compared to no alcohol consumption. The mechanism underlying the association between alcohol consumption and symptom severity in fibromyalgia requires further investigation. Future studies may consider assessing the relationship between GABA, alcohol consumption and fibromyalgia symptoms.

## Abbreviations

ANOVA: analysis of variance; ANCOVA: analysis of covariance; BMI: body mass index; FIQ: Fibromyalgia Impact Questionnaire; FTP: Fibromyalgia Treatment Program; GABA: γ-Aminobutyric Acid; HSD: honestly significant difference; SF-36: Short Form-36 Health Survey; QOL: quality of life.

## Competing interests

The authors declare that they have no competing interests.

## Authors' contributions

CHK conceived and designed the study, collected, analyzed and interpreted the data and drafted the manuscript. THO participated in the design of the study, analyzed and interpreted the data, and critically edited and revised the manuscript. AV, CAL and JMT participated in the design of the study and analysis and interpretation of the data and critically reviewed and edited the article. DJC and TDS participated in the analysis and interpretation of the data and critically evaluated the manuscript. All authors read and approved the manuscript for publication.
